# Global 3-hourly wind-wave and swell data for wave climate and wave energy resource research from 1950 to 2100

**DOI:** 10.1038/s41597-023-02151-w

**Published:** 2023-04-20

**Authors:** Xingjie Jiang, Botao Xie, Ying Bao, Zhenya Song

**Affiliations:** 1 First Institute of Oceanography, and Key Laboratory of Marine Science and Numerical Modeling, Ministry of Natural Resources, Qingdao, 266061 China; 2 Laoshan Laboratory, Qingdao, 266237 China; 3 Shandong Key Laboratory of Marine Science and Numerical Modeling, Qingdao, 266061 China; 4 China National Offshore Oil Corporation Research Institute, Beijing, 100028 China

**Keywords:** Physical oceanography, Physical oceanography

## Abstract

Ocean wave climate, including wind waves and swells, is essential to human marine activities and global or regional climate systems, and is highly related to harnessing wave energy resources. In this study, a global 3-hourly instantaneous wave dataset was established with the third-generation wave model MASNUM-WAM and wind forcings derived from the products of the First Institute of Oceanography-Earth System Model version 2.0, the climate model coupled with wave model, under the unified framework of the Coupled Model Intercomparison Project phase 6. This dataset contains 17 wave parameters, including the information associated with wave energy and spectral shape geometries, from one historical (1950–2014) simulation and three future (2015–2100) scenario experiments (ssp125, ssp245, and ssp585). Moreover, all the parameters can be accessed separately in the form of wind waves and swells. The historical results show that the simulated wave characteristics agree well with satellite observations and the ERA5 reanalysis products. This dataset can provide the community with a unique and informative data source for wave climate and wave energy resource research.

## Background & Summary

Ocean waves, arriving from specific wind events that are occurring locally (wind waves) or have occurred somewhere else on the sea surface (swells), can reach tens of meters in height or travel thousands of miles and can bring serious threats to various marine activities, such as sea voyages^1–3^, ocean fishing^4,5^, and oil exploitation^6–8^. Ocean waves can also be intimately involved in the energy and material exchange between the atmosphere and ocean, playing a crucial role in global and regional climate systems^9,10^. Therefore, understanding the wave climate and change is valuable for offshore engineering structure safety, shoreline protection, global warming prevention, etc. Moreover, harnessing wave energy (the most concentrated and high-available source of marine renewable energy with great potential for exploitation^11,12^) needs to consider its annual or seasonal spatial distributions and temporal variability, which is highly correlated with the wave climatology of the target area.

In studies on wave climate, especially in those interested in future climatic scenarios, simulation with numerical wave models is the main method used. With specific wind forcings, the simulation can generate long-term wave parameters with continuous coverage in space and time, which can be applied in further analyses. A series of wave datasets are proposed to support such research, e.g., the earlier proposed ERA-Interim^13^ provides the basic bulk wave parameters, such as significant wave height, mean wave period, and mean wave direction. The later presented ERA5 reanalysis^14–16^ and EMC/NCEP wave hindcast^17^ datasets can further exhibit those wave parameters above in the forms of wave spectral partitions, i.e., wind waves and swells, and the ERA5 dataset can also provide the parameters associated with spectral shape geometries, which can be adopted in extreme wave event analyses^18–21^. In addition to the reanalysis and hindcast datasets, wave data products from global and regional climate models can be helpful in understanding the response of the global wave climate to both historical and future climate change. For example, the First Institute of Oceanography-Earth System Model version 2.0 (FIO-ESM v2.0)^22^ was used to carry out Coupled Model Intercomparison Project phase 6 (CMIP6)^23,24^ experiments, and a global monthly and 3-hourly ocean wave dataset spanning centuries was produced^25^. Through the Coordinated Ocean Wave Climate Project (COWCLIP)^10^ phase 2, a global ensemble of ocean wave climate projections from CMIP5-driven^26^ models could be accessed^27^. Similarly, through COWCLIP2, a global ensemble of ocean wave climate statistics from contemporary reanalysis and hindcasts was also released recently^28^.

However, in those wave datasets derived from climate models, wave parameters associated with the wave energy resources, as well as the information related to shape geometries of wave spectra may be less considered, and detailed wave characters from spectral partitions are also unavailable. In this study, the wind products simulated by FIO-ESM v2.0 under the unified framework of CMIP6 were adopted again to force a standalone wave model, and then, a global 3-hourly wave dataset was generated. The dataset described here contains up to 17 wave parameters, including the characteristics associated with wave energy and spectral geometries, from one historical (1950–2014) simulation and three future (2015–2100) scenario experiments. Moreover, all the wave parameters can be accessed separately in the forms of wind waves and swells. Therefore, the newly proposed dataset differs from the other wave datasets mentioned above and can provide the community with a unique data source for wave climate and wave energy resource research.

## Methods

This dataset was established with the third-generation wave model MASNUM-WAM and wind forcings derived from the products of FIO-ESM v2.0 under the framework of CMIP6. In this section, we introduce the wave model and the wind forcings adopted in this study. Brief introductions to calculating wave parameters and identifying wind waves and swells are also presented.

### MASNUM-WAM and modeling configuration

The MASNUM-WAM (formerly LAGFD-WAM)^29–32^ is a third-generation wave model developed by the Key Laboratory of MArine Science and NUmerical Modeling (MASNUM), FIO of MNR (Ministry of Nature Resources) of China. MASNUM-WAM solves the energy spectrum balance equation in wavenumber space and uses a complicated characteristic inlaid scheme^29^ in spherical coordinates^31^ to perform shoaling and refraction effects in shallow waters, the modulation of background current to wave evolution, and the refraction of waves propagating along great circles.

In this work, the ST6 source function package^33–35^ is adopted to simulate the effects of wind input, white-capping dissipation, and swell dissipation on the evolution of waves, and the DIA^36,37^ scheme is adopted to calculate the nonlinear energy transfer between waves. A global computational grid is used in the simulation, covering the region from 80°*S* to 80°*N* and 0°(360°)*E* to 359°*E* with a 1°×1° horizontal resolution. The modeling spectral space is set as 24 directions with intervals of 15° and 35 wavenumbers spaced logarithmically from the minimum of 0.0071 up to 4.6341 with intervals of *k*_*i*+1_/k_*i*_ = 1.21, which are equivalent to frequencies from 0.042 Hz to 1.073 Hz with a ratio of 1.1 at infinite depth. Finally, bathymetric data are obtained from ETOPO1^38^ of the National Oceanic and Atmospheric Administration (NOAA) National Geophysical Data Centre (NGDC).

### FIO-ESM v2.0 and the winds from CMIP6 experiments

FIO-ESM v2.0 is a global climate model that consists of five coupled components: an atmosphere general circulation model (Community Atmosphere Model version 5^39^), a land surface model (Community Land Model version 4.0^40^), an ocean general circulation model (Parallel Ocean Program version 2^41^), an ocean surface wave model (MASNUM-WAM^29–32^), and a sea ice model (Los Alamos sea ice model version 4^42^). FIO-ESM v2.0 has considered four distinctive physical processes, including nonbreaking surface wave-induced vertical mixing^43,44^, the effects of Stokes drift on momentum and heat fluxes, the effects of sea spray on heat flux, and the SST diurnal cycle.

Recently, FIO-ESM v2.0 was used to carry out CMIP6 experiments^23^. The wind data products are derived from the CMIP6 historical data, and then, the three future scenario experiments are adopted as driving forcings in this work. The historical simulation represents climate change over the 1850–2014 period, and the future scenario experiments, which belong to the CMIP6-Endorsed Scenario Model Intercomparison Project (ScenarioMIP)^24^, are the projections of future (during 2015–2100) climate change. In this work, the three future scenarios are forced by the latest proposed shared socioeconomic pathways (SSPs), denoted ssp126, ssp245, and ssp585, representing the low, medium, and high ends of the range of future forcing pathways to produce radiative forcings of 2.6 W m^−2^, 4.5 W m^−2^, and 8.5 W m^−2^ in 2100, respectively. All four experiments mentioned above were forced by the forcing datasets provided by CMIP6 (https://esgf-node.llnl.gov/search/input4mips/).

The derived wind forcing data are the zonal and meridional wind velocities at 10 m above the sea surface. The horizontal resolution is a finite volume grid (approximately 0.9° × 1.25°), and the time resolution is three hours.

### Wave parameters

As a spectral model adopted in the simulation, wave parameters can be obtained by integrating the simulated 2-D wave spectra. The 2-D wavenumber-direction spectra simulated in MASNUM-WAM can be expressed as *E*(*k, θ*), which can be easily transformed to the frequency-direction energy spectra *S*(*f, θ*) as follows:

1 $$S\left(f,\theta \right)=\frac{1}{2\pi }{c}_{g}E\left(k,\theta \right).$$

In Eq. (1),

2 $${c}_{g}=\frac{2\pi f}{k}\left(1+\frac{2kd}{sinh\left(2kd\right)}\right)$$

is the group velocity of waves, and wavenumber *k* associated with given frequency *f* and water depth *d* (unit: m) is defined implicitly through the following dispersion relationship:

3 $${\left(2\pi f\right)}^{2}=gk\,tanh(kd).$$

Then, the one-dimensional spectrum *S*(*f*) can be obtained as follows:

4 $$S\left(f\right)={\int }_{0}^{2\pi }S\left(f,\theta \right)d\theta ,$$

and the n-th spectral moment can be expressed as follows:

5 $${m}_{n}={\int }_{0}^{\infty }{f}^{n}S\left(f\right)df.$$

The bulk wave parameters, such as the significant wave height *H*_*s*_, the wave energy period *T*_*e*_, the mean zero-crossing wave period *T*_*m*02_, and the mean wave period *T*_*m*01_, can be obtained directly via the spectral moments as follows:

6 $${H}_{s}=4\sqrt{{m}_{0}}$$

7 $${T}_{e}=\frac{{m}_{-1}}{{m}_{0}}$$

8 $${T}_{m02}=\sqrt{\frac{{m}_{0}}{{m}_{2}}}$$

9 $${T}_{m01}=\frac{{m}_{0}}{{m}_{1}}$$

The mean wave direction *θ*_*m*_ can be expressed as follows:

10 $${\theta }_{m}{=\tan }^{-1}\left(b/a\right)$$

in which

11 $$\left\{\begin{array}{c}a={\int }_{0}^{2\pi }{\int }_{0}^{\infty }\cos \theta S\left(f,\theta \right)df\,d\theta \\ b={\int }_{0}^{2\pi }{\int }_{0}^{\infty }\sin \theta S\left(f,\theta \right)df\,d\theta \end{array}\right..$$

Parameters associated with spectral shape geometries can also be obtained directly from the simulated spectra, e.g., the peak frequency *f*_*p*_ is defined as the frequency representing the maximum value of *S*(*f*); the peak wave direction *d*_*p*_ is expressed as follows:

12 $${d}_{p}{=\tan }^{-1}\left({b}_{p}/{a}_{p}\right)$$

with

13 $$\left\{\begin{array}{c}{a}_{p}={\int }_{0}^{2\pi }\cos \theta \,S\left({f}_{p},\theta \right)d\theta \\ {b}_{p}={\int }_{0}^{2\pi }\sin \theta \,S\left({f}_{p},\theta \right)d\theta \end{array}\right.;$$

The wavelength at the peak wavenumber *k*_*p*_ is calculated as follows:

14 $${L}_{p}=\frac{2\pi }{{k}_{p}}$$

and *k*_*p*_ is associated with *f*_*p*_ according to Eq. (3), and the peak wave period *T*_*p*_ is defined as the reciprocal of *f*_*p*_ and calculated using a parabolic fit around *f*_*p*_. In this work, additional geometries are also provided, such as the Goda peakedness^45^

15 $${Q}_{p}=\frac{2}{{m}_{0}^{2}}{\int }_{0}^{\infty }f{\left[{\int }_{0}^{2\pi }S\left(f,\theta \right)d\theta \right]}^{2}df,$$

the spectral bandwidth^46^

16 $${\rm{\nu }}=\sqrt{\frac{{m}_{0}{m}_{2}}{{m}_{1}^{2}}-1},$$

and the mean directional spreading^47^

17 $${\sigma }_{\theta }={\left\{2\left[1-{\left(\frac{{a}^{2}+{b}^{2}}{{m}_{0}^{2}}\right)}^{1/2}\right]\right\}}^{1/2}$$

(where *a* and *b* are obtained according to Eq. (11)).

Moreover, wave parameters, which can be applied to wave energy assessment and characterization, are also presented. According to the Technical Specification proposed by the International Electrotechnical Commission^48^ (IEC TS 62600-101:2015; hereafter IEC2015), the (omni-directional) wave power density (WPD) can be estimated as follows:

18 $$WPD=\rho g{\int }_{0}^{2\pi }{\int }_{0}^{\infty }{C}_{g}S\left(f,\theta \right)dfd\theta $$

where *ρ* (taken as 1023 *kg*/*m*^3^ in this work) denotes the density of seawater, *g* = 9.81 *m*/*s*^2^ is the acceleration of gravity, and *C*_*g*_ is the group velocity of waves in Eq. (2). IEC2015 also recommends directionally resolved WPD, i.e., resolving omni-directional WPD in a specific direction *θ*_*j*_:

19 $$WP{D}_{{\theta }_{j}}=\rho g{\int }_{0}^{2\pi }{\int }_{0}^{\infty }{C}_{g}S\left(f,\theta \right)\cos \left(\theta -{\theta }_{j}\right)\delta dfd\theta ,\left\{\begin{array}{c}\delta =1,\cos \left(\theta -{\theta }_{j}\right)\ge 0\\ \delta =0,\cos \left(\theta -{\theta }_{j}\right) < 0\end{array}\right..$$

In this work, *θ*_*j*_ are assigned to the 24 discrete directions in the spectral space, and the maximum value of $WP{D}_{{\theta }_{j}}$ (denoted as $WP{D}_{{\theta }_{j},max}$) and the corresponding direction *θ*_*j,max*_ are retained for each simulated spectrum. Furthermore, to measure the relative spread of wave energy in the *f* and *θ* directions, IEC2015 recommends the following coefficients:

20 $${\epsilon }_{0}=\sqrt{\frac{{m}_{0}{m}_{-2}}{{m}_{-1}^{2}}-1}$$

and

21 $${d}_{\theta }=\frac{WP{D}_{{\theta }_{j},max}}{WPD},$$

respectively.

Finally, the wind-sea fraction (WSF) parameter is introduced to characterize the proportion of wind wave energy contained in each spectrum and is presented as follows:^49,50^

22 $$WSF=\frac{{E}_{{U}_{p} > c}}{{m}_{0}}$$

where ${E}_{{U}_{p} > c}$ is the energy in the spectral space for which the projected wind speed *U*_*p*_ is larger than the local wave phase velocity *c*. The parameter *U*_*p*_ can be calculated as follows:

23 $${U}_{p}={C}_{mult}{U}_{10}{\cos }(\delta ),$$

where *U*_10_ denotes the wind speed at the height of 10 m above the sea surface, *δ* denotes the angle between the direction in the spectral space and the direction in which the wind is blowing, and *C*_*mult*_ is a coefficient set as 1.7 in this work. In the spectral space, wave phase velocity *c* is associated with frequency *f* and wavenumber *k*; thus, according to Eq. (3),

24 $$c=\frac{2\pi f}{k}=\sqrt{\frac{g}{k}}\cdot \sqrt{\tanh \left(kd\right)}.$$

### Identification of wind waves and swells

The spectral partitioning technique was introduced to demonstrate the historical and future wave characteristics in the forms of wind waves and swells. The spectral partitioning technique can be traced back to a digital image processing watershed algorithm^51^, which can be adopted to identify watershed lines, mountain peaks, and valleys in topographic maps. Because the 2D spectrum resembles a topological surface, it is logical to apply such an algorithm in this circumstance^52^. As described by Hanson and Phillips^49^, the basic approach to the spectral partitioning method is that by searching through the spectral matrix *S*(*f, θ*), the paths of steepest ascent leading to each peak or local energy maximum can be identified; then, all paths leading to the same peak can be grouped, and the members that lie on the collection of the paths are considered to belong to a distinct partition. Partitioning of wave spectra is widely adopted in research concerning data assimilation^52–54^, spatial and temporal tracking of wave systems^49,55^, and so on.

Notably, the wave parameters mentioned above can be calculated not only from the entire spectrum but also from a partition of it. In each partitioned spectrum, partitions whose *WSF* are greater than 33.33%, together with spectral elements (*f, θ*) whose phase velocities (Eq. (24)) are less than the local projected wind speeds (Eq. (23)), are combined as a new partition, and the newly formed partition is considered to be under the direct influence of the wind and thus is identified as the wind wave system; the remaining partitions (*WSF* < 33.33%), including those incomplete partitions that have contributed some elements to the wind wave system, are then identified as individual swells. The swells in the same spectrum can be combined as a total swell partition.

The partitioning and identification program implemented in this work was developed based on the W3PARTMD module of WaveWatch III ver. 6.07^56^, in which an efficient FORTRAN routine was transformed from the MATLAB code^57,58^ that was used to apply the watershed algorithm^51^. The wave parameters provided in this dataset are calculated from both the entire spectra and its wind wave and swell partitions; see the Data Records section for more details.

## Data Records

This dataset consists of up to 17 kinds of wave parameters, and wind speeds at 10 meters above the sea surface (toward the east and north) are also provided. The dataset covers the area of 0°*E*–359°*E*, 80° S–80° N with spatial intervals of 1°, and the temporal intervals are 3 hours. The 17 wave parameters presented in this dataset are integrated from both the entire spectra (i.e., combined wind waves and swells, hereafter COMB) and their partitions, and the partitions are presented in the forms of wind waves (WSEA), total swells (TSWL), and the first three swells with the largest *H*_*s*_ (SWL1, SWL2, and SWL3). Moreover, the wave dataset spans a 65-year historical period (1950–2014) and three 86-year future scenarios (2015–2100).

The data mentioned above are stored monthly for each wave and wind parameter, and the filenames of the data are in the following format:

<para_id>_<exp_id>_<yyyymm>.nc,where <para_id> denotes the name of parameters, see Table 1; <exp_id> represents the name of the CMIP6 driving conditions, which are ‘histor’, ‘ssp126’, ‘ssp245’, and ‘ssp585’; and <yyyymm> are expressed as 195001–201412 for the ‘histor’ data and as 201501–210012 for the three future scenarios. Since there are 780 and 1032 months during 1950–2014 and 2015–2100, respectively, and there are 17 wave parameters and 2 wind parameters to be exhibited, the number of files in the historical catalog is 780 × 19 = 14820, and the number of files for each future scenario is 1032 × 19 = 19608.

**Table 1:** List of all variables in the dataset.

No.	<para_id>	Description	Dimensions	Units
1	Hs	Significant wave height	[lon, lat, npt, time]	m
2	Tp	Peak wave period	[lon, lat, npt, time]	s
3	Te	Wave energy period	[lon, lat, npt, time]	s
4	Tm01	Mean wave period	[lon, lat, npt, time]	s
5	Tm02	Mean zero-crossing period	[lon, lat, npt, time]	s
6	fp	Peak frequency	[lon, lat, npt, time]	Hz
7	Lp	Peak wavelength	[lon, lat, npt, time]	m
8	Dirm	Mean wave direction (Cartesian To)	[lon, lat, npt, time]	degr.
9	Dirp	Peak wave direction (Cartesian To)	[lon, lat, npt, time]	degr.
10	Spr	Mean directional spreading^47^	[lon, lat, npt, time]	degr.
11	nu	Spectral bandwidth^46^	[lon, lat, npt, time]	—
12	Qp	Goda peakedness^45^	[lon, lat, npt, time]	—
13	WSF	Wind sea fraction	[lon, lat, npt, time]	%
14	WPD	Wave power density^48^	[lon, lat, npt, time]	kW/m
15	WPeps0	Relative spread of wave energy in frequency dimension^48^	[lon, lat, npt, time]	—
16	WPthmx	Maximum value of directionally resolved WPD^48^	[lon, lat, npt, time]	kW/m
17	thWPmx	Direction of WPthmx (Cartesian To)^48^	[lon, lat, npt, time]	degr.
19	windx	Wind speed toward east	[lon, lat, time]	m/s
20	windy	Wind speed toward north	[lon, lat, time]	m/s

All data files are provided in NetCDF format and are archived in the ScienceDB^59^. The variables in each file, together with their descriptions, dimensions, and units, are outlined in Table 1. The dimension of time is presented as days since 1950-01-01 00:00:00; the dimensions of latitude and longitude are expressed as degrees north and east, respectively; and variables associated with waves contain the ‘npt’ dimension, and npt from 1 to 6 indicate variables derived from the COMB, WSEA, TSWL, and SWL1-3, respectively. In this dataset, ‘_FillValue’ denotes land points; in particular, wave-associated variables may present a negative value, indicating that the spectrum or the spectral partition from which the parameter is obtained contains much less energy, such that the corresponding *H*_*s*_ is less than 0.05 m.

## Technical Validation

The MASNUM-WAM has been calibrated and adopted many times in previous scientific and engineering studies (e.g.^60–65^); moreover, MASNUM-WAM is now the ocean wave component of several operational ocean forecasting systems (OFS), such as the OFS for the seas of China and adjacent areas^66^, OFS for Southeast Asian Seas and OFS for the 21st-Century Maritime Silk Road^67^. Therefore, validation of the MASNUM-WAM is not shown in this study.

The validation of FIO-ESM v2.0 can be referred to in the work of Bao *et al*.^22^, in which FIO-ESM v2.0 was applied to conduct the CMIP6 DECK (Diagnostic, Evaluation and Characterization of Klima) and historical (1850–2014) experiments^23^. The results show that the time evolutions of surface air temperature, sea surface temperature, and Atlantic meridional overturning circulation in the past centuries are well reproduced; in particular, the common, large, warm sea surface temperature bias for all climate models is dramatically reduced, and the simulated El Niño-Southern Oscillation period is much closer to the observation within 2–7 years. Therefore, it is suggested that the performance of FIO-ESM v2.0 under the CMIP6 experimental framework is stable and reliable, including in both the historical and future scenarios. Moreover, Song *et al*.^25^ performed the validation of the FIO-ESM v2.0 wave product in the CMIP6 historical experiment. In the comparison against the ERA5 reanalysis data from 1979–2014, the monthly mean *H*_*s*_, *θ*_*m*_, *T*_*p*_, and *T*_*m*02_ show good agreement in terms of the basic characteristics of spatial pattern and seasonal variation, as well as the 99th-percentile values of *H*_*s*_ derived from the 3-hourly data.

As the aim of this dataset is to aid in wave climate and wave energy resource research, and as most of the research characteristics of interest are closely related to the parameters of *H*_*s*_ and *T*_*e*_, the validation focuses on the climatology of the two key parameters, denoted as Hs-MAS and Te-MAS, in this dataset. In addition, the quality of wind forcing adopted to force MASNUM-WAM is also to be validated, and we focus on the wind speed (WS), denoted as WS-MAS, in this section.

The AVISO gridded wind and wave products^68^ are selected as the observation baseline to be compared with WS-MAS and Hs-MAS. The AVISO products comprise the daily WS and *H*_*s*_ observations, denoted as WS-OBS and Hs-OBS, respectively, merged from a set of missions, such as Envisat, Jason-1–3, AltiKa, and Sentinel-3A, and it covers 0°*E* to 359°*E*, 90°*S* to 89°*N* with a horizontal resolution of 1° × 1°. Notably, to be comparable with the MASNUM-WAM simulated results in the historical scenario, the sampling periods are 2013–2014 and 2010–2014 for WS-OBS and Hs-OBS, respectively. To validate WS-MAS with a longer sampling period and to validate Hs-MAS and Te-MAS in the forms of wind waves and swells separately, the ‘ERA5 hourly data on single levels from 1959 to present’ dataset^69^ is adopted. The ERA5 hourly data can provide 10m u-v winds and separate COMB, WSEA, and TSWL wave characteristics, covering 0°*E*–360°*E*, 90°*S*–90°*N* with spatial intervals of 0.25° and 0.5° for the wind and wave parameters, respectively; to be comparable with Hs-MAS and Te-MAS, the original spatial and temporal resolutions are reduced and the sampling period is selected as 2010–2014. Then, the employed ERA5 WS, *H*_*s*_ and *T*_*e*_ products are denoted as WS-ERA, Hs-ERA and Te-ERA, respectively. Finally, to perform a more general validation, a global ensemble of ocean wave climate statistics^28^ is introduced. As a product of the COWCLIP2^10,28^, the statistics mentioned above comprise 14 contemporary wave reanalysis and hindcasts computed across 1980–2014, including general and extreme statistics of *H*_*s*_, mean wave period (such as *T*_*m*01_ and *T*_*m*02_), and *θ*_*m*_ at different frequency resolutions (monthly, seasonally, and annually). We employed the mean values of annually averaged *H*_*s*_, denoted as Hs-COW, in the statistics as the baseline to validate the historical Hs-MAS. Similarly, the spatial resolution of Hs-COW has been adjusted to that of Hs-MAS. Furthermore, in the comparisons below, four quantitative errors are also exhibited: the Pierson’s correlation coefficient R

25 $$R=\frac{{\sum }_{i}(({S}_{i}-\bar{S})({O}_{i}-\bar{O}))}{\sqrt{{\sum }_{i}{({S}_{i}-\bar{S})}^{2}\cdot {\sum }_{i}{({O}_{i}-\bar{O})}^{2}}},$$

the mean absolute error MAE

26 $${\rm{MAE}}=\frac{1}{N}{\sum }_{i=1}^{N}| {S}_{i}-{O}_{i}| ,$$

the mean bias B

27 $$B=\frac{1}{N}{\sum }_{i=1}^{N}\left({S}_{i}-{O}_{i}\right),$$

and the root-mean-square error RMSE

28 $$RMSE=\sqrt{\frac{1}{N}{\sum }_{i=1}^{N}{\left({S}_{i}-{O}_{i}\right)}^{2}}.$$

In Eqs. (25–28), *S* denotes the key parameters from the descripted dataset, i.e., WS-MAS, Hs-MAS or Te-MAS, and *O* indicates the corresponding parameters from AVISO observations, ERA5 reanalysis, or COWCLIP2 products. Notably, the abovementioned *S* and *O* represent the annual-averaged statistics or the 95-th percentiles over the time in each dataset to be compared. Thus, the quantitative comparisons with Eqs. (25–28) are performed on space, i.e., the subscript *i* indicates the index of each water grid point in *S* and *O*, considering the total number of sampling grid points *N* and $\bar{S}=\frac{1}{N}{\sum }_{i=1}^{N}{S}_{i}$ and $\bar{O}=\frac{1}{N}{\sum }_{i=1}^{N}{O}_{i}$. The spatial resolutions of *S* and *O* are unified as mentioned previously.

### Comparisons of WS-MAS against WS-OBS and WS-ERA

**Fig. 1:**
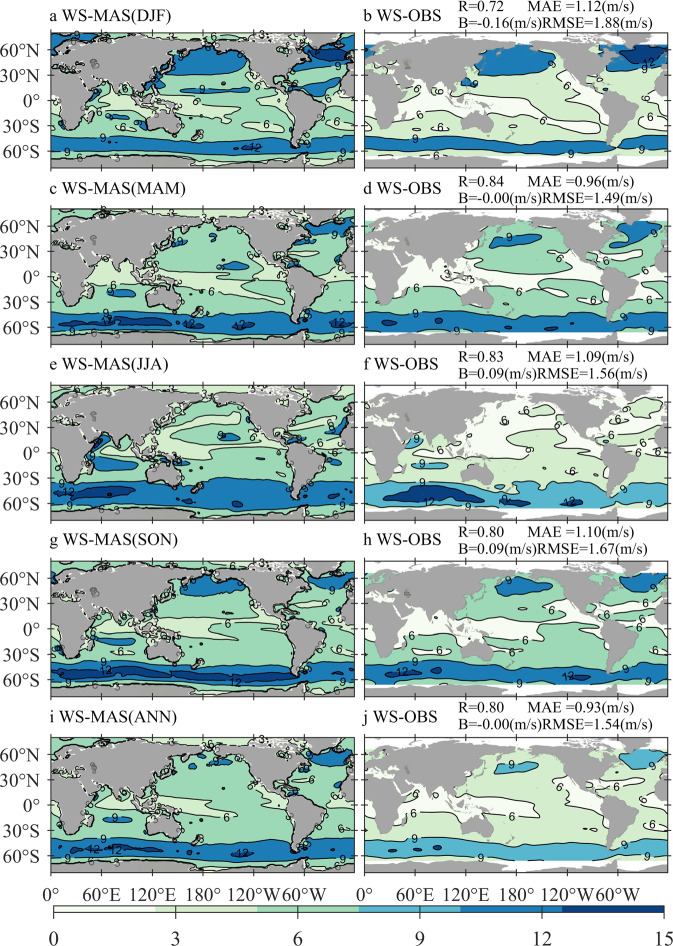
Climatological distributions of the averaged WS-MAS (wind speed adopted to force MASNUM-WAM, left column) and WS-OBS (wind speed from observation, right column) in boreal winter (**a**,**b**), boreal spring (**c**,**d**), boreal summer (**e**,**f**), boreal autumn (**g**,**h**), and 2013–2014 (**i**,**j**), with the corresponding quantitative errors exhibited in panels b, d, f, h, and j, respectively.

Comparisons of WS-MAS against WS-OBS and WS-ERA can validate the quality of the forcing wind. Figure 1 illustrates the climatological distributions of WS-MAS and WS-OBS in boreal winter (December-January-February, DJF, panels a,b), spring (March-April-May, MAM, panels c,d), summer (June-July-August, JJA, panels e–f), and autumn (September-October-November, SON, panels g–h) during 2013–2014, as well as the annual (ANN, panels i–j) mean result. The quantitative errors between the seasonal- or annual-averaged WS-MAS and WS-OBS are also exhibited in the same rows of the corresponding panels. Figure 1 shows that WS-WAM and WS-OBS can achieve a strong level of agreement around the world. The distribution patterns of the two characteristics are quite similar for both seasonal and annual mean statistics. The mean value of quantitative error B can even be 0.00 m/s when all the samples are involved.

**Fig. 2:**
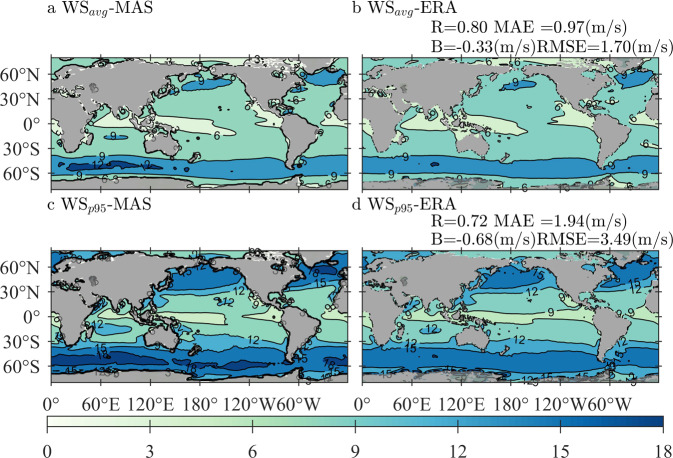
Climatological distributions of (**a**) WS_avg_-MAS (averaged wind speed from the forcings of MASNUM-WAM), (**b**) WS_avg_-ERA (averaged wind speed from ERA5), (**c**) WS_p95_-MAS (95th-percentile wind speed from the forcings of MASNUM-WAM) and (**d**) WS_p95_-ERA (95th-percentile wind speed from ERA5), with the corresponding quantitative errors exhibited in panels b and d, respectively. The sampling period is from 2010 to 2014.

The climatological distributions of averaged WS-MAS and WS-ERA (panels a–b), together with the 95-th percentile of the two characters (panels c–d), are illustrated in Fig. 2, with the corresponding quantitative errors exhibited in panels b and d, respectively. The sampling period is from 2010 to 2014. As shown in Fig. 2, the distribution patterns of WS-MAS and WS-ERA can match strongly in both mean and extreme conditions, although understandably, the quantitative errors in extreme conditions are slightly higher than those in mean conditions.

Therefore, we can conclude that the quality of the forcing wind derived from the FIO-ESM v2.0 product is robust and reliable.

### Comparison between Hs-MAS and Hs-OBS

**Fig. 3:**
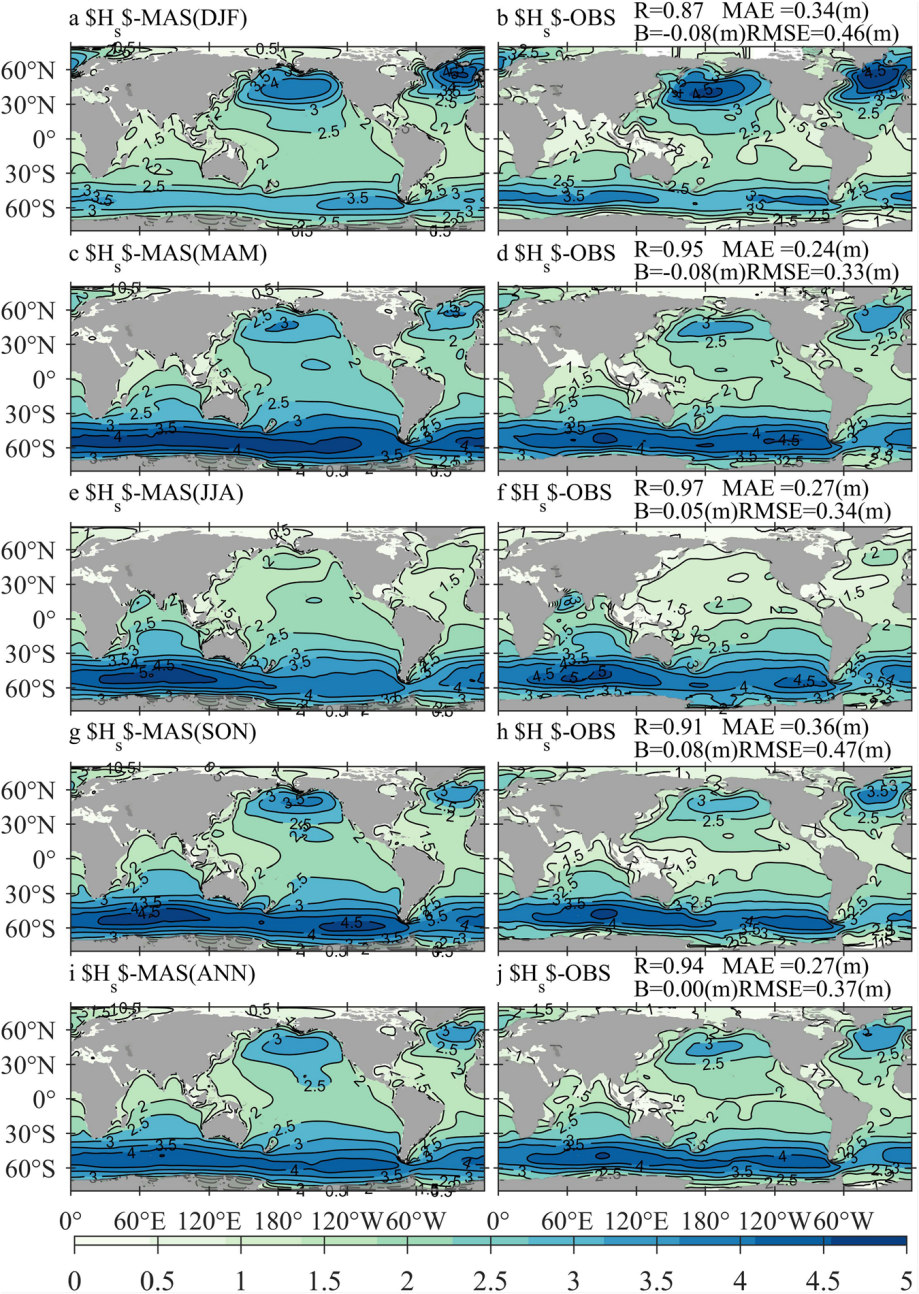
Climatological distributions of the averaged Hs-MAS (significant wave height simulated by MASNUM-WAM, left column) and Hs-OBS (significant wave height from observation, right column) in boreal winter (**a**,**b**), boreal spring (**c**,**d**), boreal summer (**e**,**f**), boreal autumn (**g**,**h**), and 2010–2014 (**I**,**j**), with the corresponding quantitative errors exhibited in panels b, d, f, h, and j, respectively.

A comparison between Hs-MAS and Hs-OBS can be used to assess the seasonal and annual mean state of the simulated *H*_*s*_ in spatial distribution patterns. The climatological distributions of averaged Hs-MAS and Hs-OBS in boreal winter (December-January-February, DJF, panels a,b), spring (panels c,d), summer (panels e,f), and autumn (panels g,h) during 2010–2014, together with the annual (panels i,j) mean result, are illustrated in Fig. 3. The quantitative errors between the seasonal- or annual-averaged Hs-MAS and Hs-OBS are also exhibited in the same rows of the corresponding panels.

Figure 3 shows that the comparison against the satellite-observed *H*_*s*_ has a good agreement. The seasonal- and annual-averaged Hs-MAS and Hs-OBS distribution patterns are very similar. The differences are generally restricted within ±0.4 m in most oceans around the world for the three-monthly averages and are even reduced to ±0.2 m when the yearly mean values are considered. From the quantitative errors, the MAE and RMSE are generally less than 0.3 m and 0.4 m, respectively, the values of the R coefficients are generally greater than 0.9, and all the B values are very close to 0 m.

Although the MASNUM-WAM simulations with the FIO-ESM v2.0 wind forcings can capture the basic characteristics of ocean waves, large differences from observations in some sea areas are still inevitable. For example, Hs-MAS is larger than Hs-OBS by approximately 0.4–0.8 m in the seas south of 60°*S* throughout the years averaged, and the extreme value can be over 1.2 m in DJF and SON. In contrast, Hs-MAS is smaller than Hs-OBS in the Arctic Ocean, and extreme differences of −0.8 m can be found in DJF. Moreover, Hs-MAS is smaller than Hs-OBS in all four seasons over the North Atlantic, where the absolute differences are smaller in JJA and larger in DJF, with values of approximately 0.2–0.4 m and 0.8–1.0 m, respectively.

### Comparisons of Hs-MAS and Te-MAS against Hs-ERA and Te-ERA

**Fig. 4:**
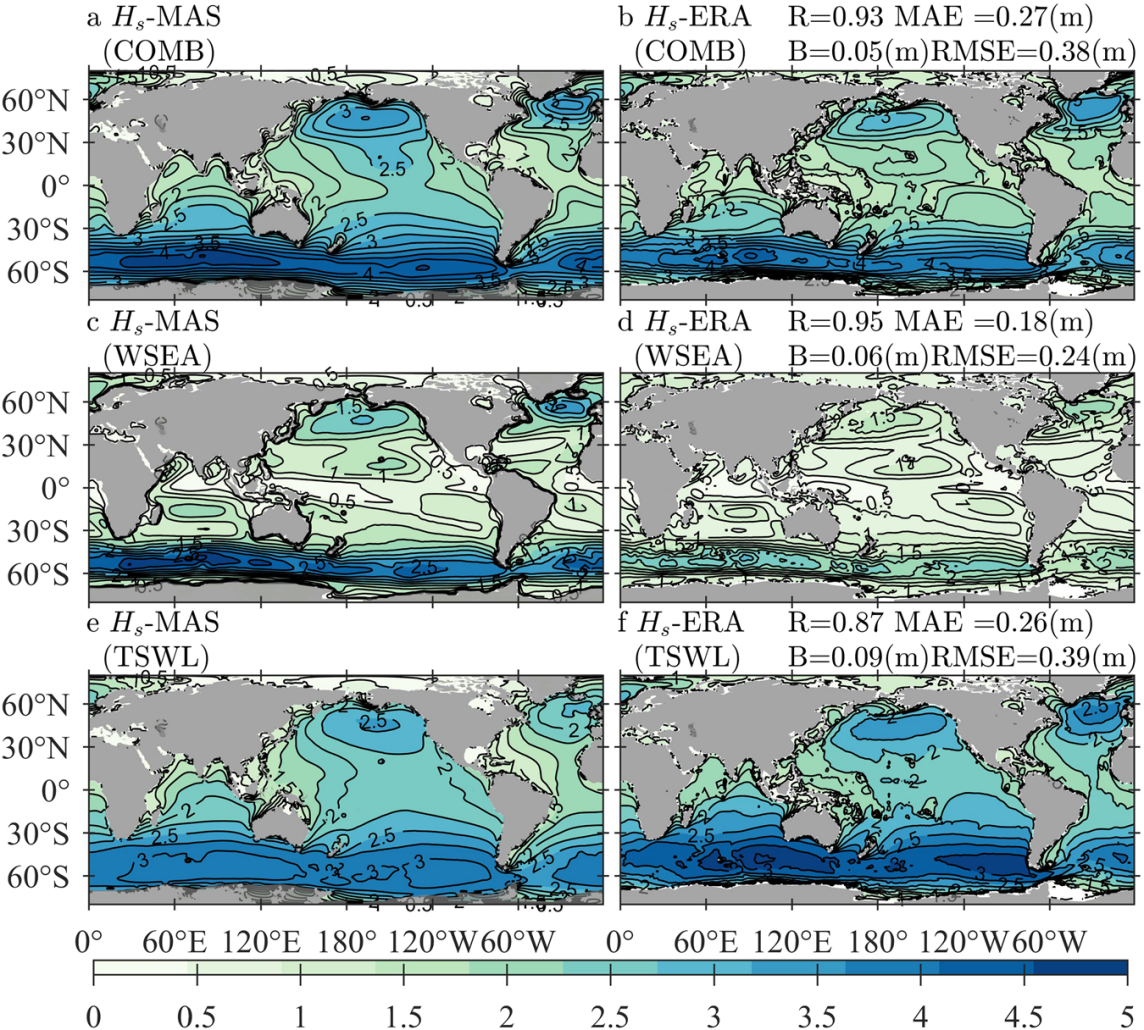
Climatological distributions of the averaged Hs-MAS (significant wave height simulated by MASNUM-WAM, left column) and Hs-ERA (significant wave height from ERA5, right column) for COMB (combined wind waves and swell, **a**,**b**), WSEA (wind waves, **c**,**d**), and TSWL (total swells, **e,f**) wave patterns, with the corresponding quantitative errors exhibited in panels b, d, and f, respectively. The average period is from 2010 to 2014.

**Fig. 5:**
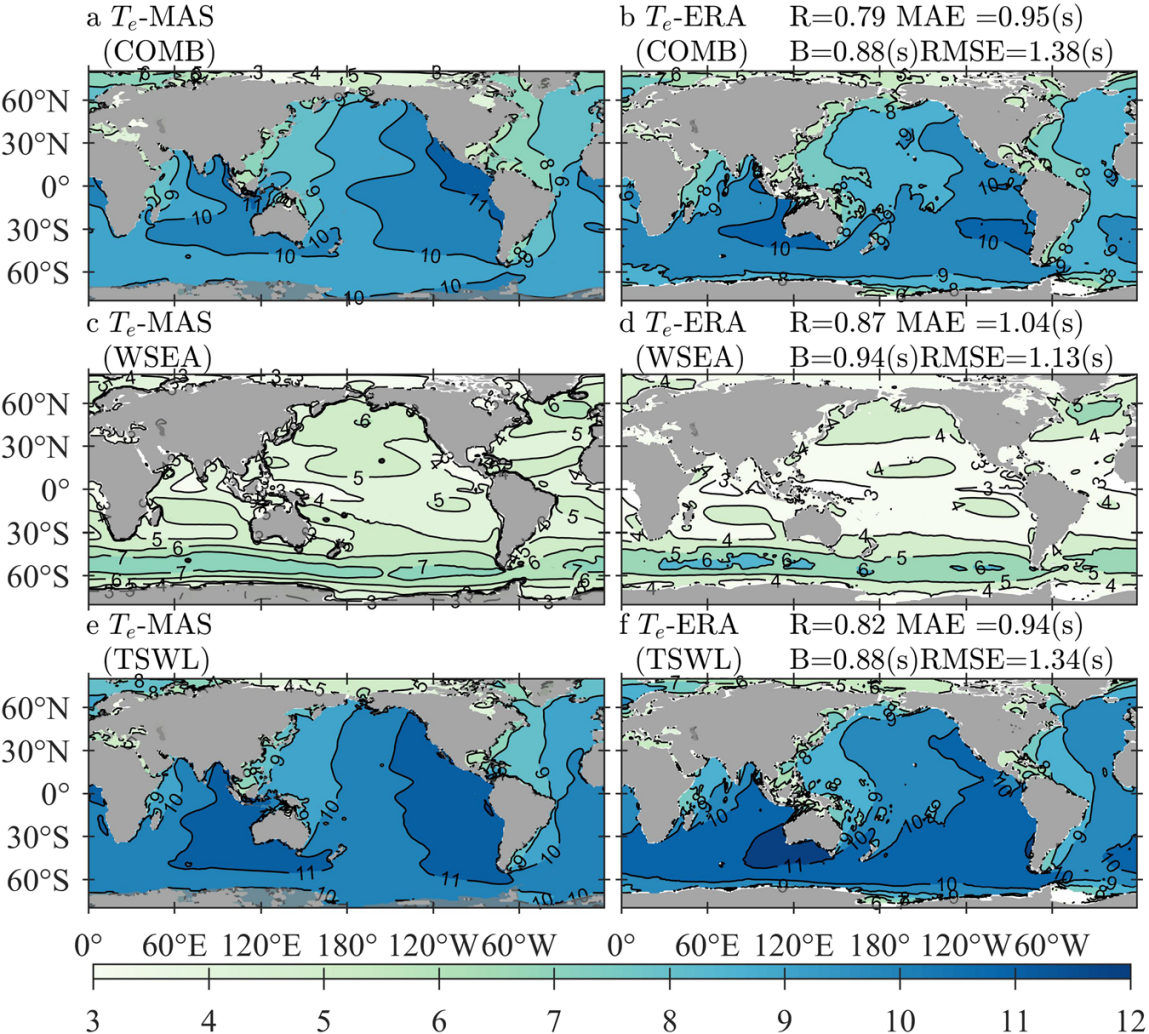
Climatological distributions of the 95-th percentile Hs-MAS (significant wave height simulated by MASNUM-WAM, left column) and Hs-ERA (significant wave height from ERA5, right column) for COMB (combined wind waves and swell, **a,b**), WSEA (wind waves, **c,d**), and TSWL (total swells, **e,f**) wave patterns, with the corresponding quantitative errors exhibited in panels b, d, and f, respectively. The sampling period is from 2010 to 2014.

Comparisons of Hs-MAS and Te-MAS against Hs-ERA and Te-ERA demonstrate the performance of the simulated *H*_*s*_ and *T*_*e*_ in the forms of wind waves and swells separately. Figure 4 illustrates the climatological distributions of annually averaged Hs-MAS and Hs-ERA in the forms of COMB (panels a,b), WSEA (panels c,d), and TSWL (panels e,f), with the quantitative errors exhibited in the same rows of the corresponding panels. In addition to the mean state, extreme conditions, i.e., the 95th-percentile values of Hs-MAS and Hs-OBS derived from 2010–2014, are presented in Fig. 5, where the panels and quantitative errors are arranged similarly to those in Fig. 4.

Figure 4 shows that the mean states of COMB Hs-MAS coincide well with those of COMB Hs-ERA, where the four errors are close to those exhibited in the comparison between the annual mean Hs-MAS and Hs-OBS. The consistency of the two datasets in WSEA is even better, but a larger difference can be found when considering TSWL conditions; nevertheless, quantitative errors between TSWL Hs-MAS and TSWL Hs-ERA still suggest acceptable goodness of fit. For extreme waves shown in Fig. 5, the comparisons also exhibit good agreements; the R coefficients continue at the high levels that have been found in Fig. 4, and the values of B, MAE and RMSE in Fig. 5 become larger due to higher wave heights involved in the statistical procedure. Differences in the spatial distribution of both Figs. 4, 5 are similar to Fig. 3. Hs-MAS is larger than Hs-ERA in the seas south of 60°*S* for both WSEA and TSWL conditions, and TSWL Hs-MAS is even larger than TSWL Hs-ERA by approximately 0.6–0.8 m in both mean and extreme conditions. In the Arctic Ocean, Hs-MAS is smaller than Hs-ERA, mainly due to the smaller estimated TSWL of Hs-MAS. In the North Atlantic, Hs-MAS is generally smaller than Hs-ERA for the mean state, but for extreme conditions, the former is larger than the latter in the eastern part of the ocean.

**Fig. 6:**
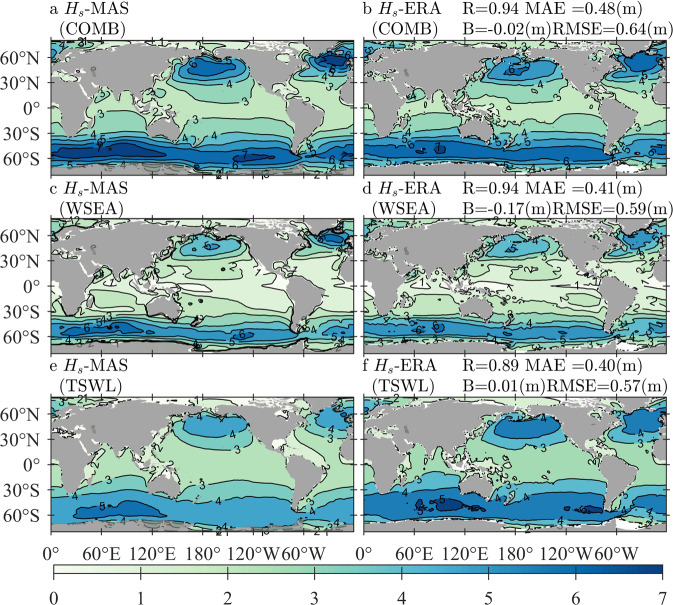
Climatological distributions of the averaged Te-MAS (wave energy period simulated by MASNUM-WAM, left column) and Te-ERA (wave energy period from ERA5, right column) for COMB (combined wind waves and swell, **a,b**), WSEA (wind waves, **c,d**), and TSWL (total swells, **e,f**) wave patterns, with the corresponding quantitative errors exhibited in panels b, d, and f, respectively. The average period is from 2010 to 2014.

**Fig. 7:**
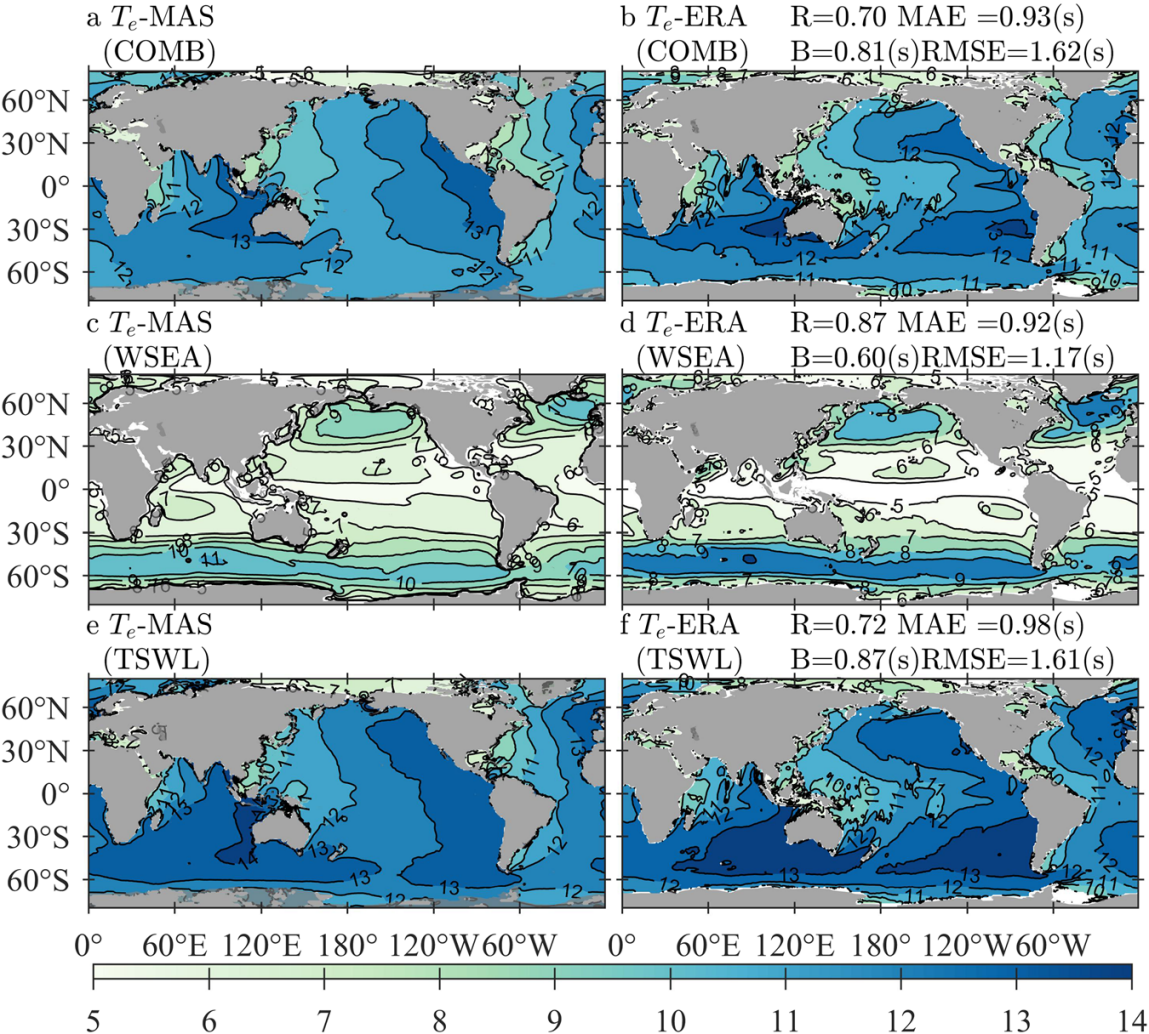
Climatological distributions of the 95th-percentile Te-MAS (wave energy period simulated by MANUM-WAM, left column) and Te-ERA (wave energy period from ERA5, right column) for COMB (combined wind waves and swell, **a,b**), WSEA (wind waves, **c,d**), and TSWL (total swells, **e,f**) wave patterns, with the corresponding quantitative errors exhibited in panels b, d, and f, respectively. The sampling period is from 2010 to 2014.

The comparisons between Te-MAS and Te-ERA for the mean and extreme conditions are shown in Figs. 6, 7, respectively. Panels for the wave patterns of COMB, WSEA, and TSWL are illustrated in the same way as Figs. 4, 5, as well as the quantitative errors. Parameters associated with wave periods may be influenced markedly by spectral shapes; thus, the simulated *T*_*e*_ in the two datasets might not easily achieve consistency, especially when spectral partitioning is conducted. Figure 6 shows that Te-MAS is larger than Te-ERA in almost all oceans around the world, including both WSEA and TSWL patterns, but the extreme bias is no more than 1.8 s. TSWL Te-MAS is smaller than TSWL Te-ERA by approximately 0.2–0.4 s in the North Atlantic, resulting in a smaller COMB Te-MAS in the same location. For the extreme conditions shown in Fig. 7, the differences between Te-MAS and Te-ERA are reduced for both WSEA and TSWL conditions, although the Te-MAS is still larger than the Te-ERA by approximately 1 s; it is noted that Te-MAS is estimated to be smaller than Te-ERA in the North Atlantic for both WSEA and TSWL wave patterns.

### Comparisons between Hs-MAS and Hs-COW

**Fig. 8:**
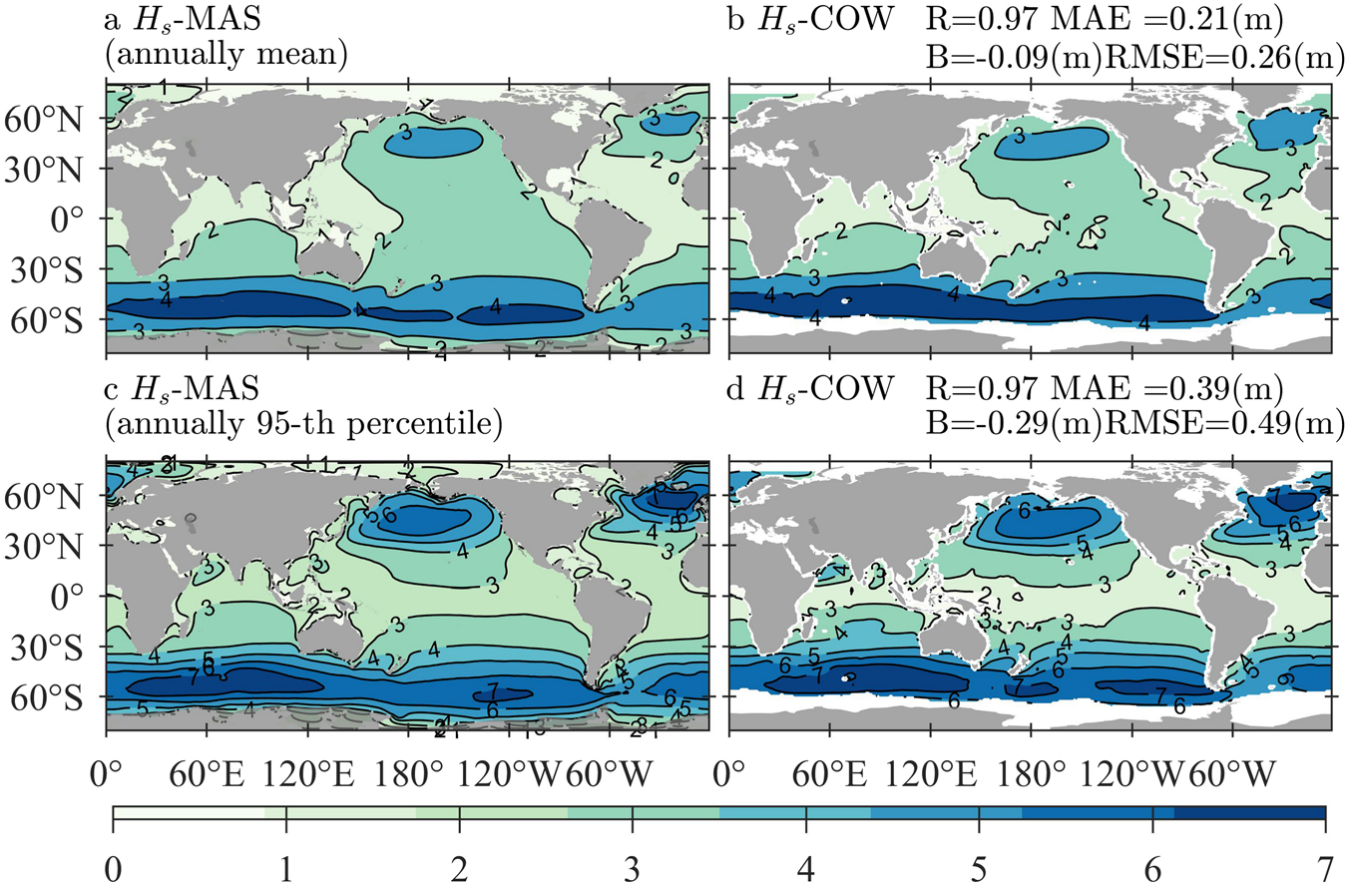
Climatological distributions of the averaged Hs-MAS (significant wave height simulated by MASNUM-WAM, left-column) and Hs-COW (significant wave height derived from COWCLIP2 dataset, right-column) for the annual mean (panels **a,b**) and 95-th percentile (panels **c,d**) statistics, with the corresponding quantitative errors exhibited in panels b and d, respectively. The sampling period is from 1980 to 2014.

Figure 8 illustrates the climatological distributions of the averaged Hs-MAS (left-column) and Hs-COW (right-column) for the annual mean (panels a–b) and 95-th percentile (panels c–d) statistics. The corresponding quantitative errors are exhibited in panels b and d, respectively.

Good agreement between the statistics of Hs-MAS and Hs-COW can still be found in Fig. 8. The deviations in spatial distribution for both mean and extreme conditions are very similar to the corresponding panels in Figs. 4, 5, as well as the quantitative errors. In addition, Hs-MAS is also smaller than Hs-COW in the North Atlantic by approximately 0.4 m for the annual mean values and by approximately 1.0 m when the 95-th percentiles are considered.

Overall, the above analyses indicate that the mean state of WS, *H*_*s*_, and *T*_*e*_ proposed in the newly established dataset can capture the basic characteristics of the satellite observations in seasonal and annual spatial distributions and can also be broadly consistent with the ERA5 products in both forms of WSEA and TSWL and both mean and extreme wave conditions. The comparisons against the more general wave statistics produced through COWCLIP2 confirm the conclusions mentioned above. However, the simulated *H*_*s*_ and *T*_*e*_ may still suffer biases, especially in the southern 60°*S*, Arctic, and North Atlantic Oceans.

## Data Availability

The source code of MASNUM-WAM is available to the public and can be downloaded from 10.57760/sciencedb.02893^70^. The dataset can be regenerated by using the wind parameter files, i.e., files with <para_id> of ‘windx’ and ‘windy’, archived in ScienceDB^59^ as wind forcings.
